# Research Status Quo in Traditional Mongolian Medicine: A Bibliometric Analysis on Research Documents in the Web of Science Database

**DOI:** 10.1155/2021/5088129

**Published:** 2021-12-28

**Authors:** Tae-Hun Kim, Jung Won Kang

**Affiliations:** ^1^Korean Medicine Clinical Trial Center, College of Korean Medicine, Kyung Hee University, 23 Kyungheedae-ro, Dongdaemun-gu, Seoul 02447, Republic of Korea; ^2^Department of Acupuncture & Moxibustion, College of Korean Medicine, Kyung Hee University, 23 Kyungheedae-ro, Dongdaemun-gu, Seoul 02447, Republic of Korea

## Abstract

**Objective:**

In this study, the current state of research on traditional Mongolian medicine (TMM) through a bibliometric analysis of research documents located in the Web of Science (WoS) database was assessed.

**Methods:**

The WoS database was searched on September 2021 with the keywords “traditional Mongolian medicine.” Publications on TMM scientific research were included in this study, without any language limitations. Bibliometric data from such publications were retrieved from the WoS database. Full records with cited reference lists were descriptively analyzed. To assess trends in TMM research topics, authors' keywords were analyzed. A thematic evolution map based on coword analysis was suggested. To analyze research networks among co-authors, affiliations, or countries of the authors, collaboration networks were evaluated. The Bibliometrix R package (3.1) was used for the analysis.

**Results:**

A total of 234 scientific publications were included in the analysis. The top three countries of origin of the corresponding authors were China (*n* = 153), Japan (*n* = 28), and South Korea (*n* = 9). The top three relevant affiliations of the authors in the included publications were “Inner Mongolia Medical University,” “Inner Mongolia University of Nationalities,” and “National University of Mongolia.” “Flavonoids,” “cytotoxicity,” “NMR,” and “Tibetan medicine” were the most frequently used keywords in the included documents. Most publications focused on the chemical analysis and mechanism of effects of Mongolian herbal medications. There were few publications on nonpharmacological interventions such as bloodletting or TMM diagnostics, which should be promoted in future publications.

**Conclusion:**

There were only a limited number of publications on TMM identified through a search of the WoS database, using the keywords “Traditional Mongolian medicine.” More improved strategy for searching for TMM publications must be established. Research publications on TMM, especially regarding nonpharmacological interventions, need to be promoted. In addition, collaboration with researchers worldwide needs to be encouraged in the future.

## 1. Introduction

Traditional medicine is defined as a system of medicine established based on specific cultural beliefs that pass on from generation to generation [1]. It is usually classified as a complementary health approach, but is used in clinical practice as mainstream medicine in some countries [2]. Research on traditional medicine can provide evidence on the effectiveness and safety of its interventions in the local areas where it is used and offers guidance on novel drug development processes to global pharmaceutical companies [3]. In this sense, research publications on traditional medicine must be identifiable and accessible outside the local region.

Bibliometrics is a subfield of research in information science and can be defined, according to Paul Otlet, as the measurement and analysis of relevant items around published books or documents [4]. Along with the contents of the research publications, metadata such as authors, institutions, year of publication, and reference lists are the subjects of interest in bibliometric analysis. To study research trends and collaboration in a specific field, these data are analyzed using mathematical calculation methods, and the results are generally suggested as figures or network graphs, which provide a basic clue as the first step to understanding the current research status in the corresponding field [5].

Traditional Mongolian medicine (TMM) is assumed to have over a 5,000-year-long history, and it has specific features such as bone art, balneotherapy, and DOM therapy developed and practiced by the Mongolian nomads, along with the adoption of extrinsic medical theory and remedies from abroad, such as Tibetan Medicine, Ayurveda, or traditional Chinese medicine (TCM) [6]. However, research on TMM is not well known outside of Mongolia, and there is not enough information to understand the current research status through the eyes of foreign researchers. In the PubMed, MeSH term for TMM, “Medicine, Mongolian Traditional” was introduced in 2010, but only a small number of literature are found using this term, which reflects the current information deficiency [7]. From this viewpoint, a brief bibliometric analysis of TMM through a search of the Web of Science (WoS) database was conducted to evaluate the current research status of TMM.

## 2. Methods

### 2.1. Literature Search and Selection of the Literature

For this study, the literature on TMM was identified through an electronic database search. The WoS, which includes extensive research literature with detailed information on the authors, institutions, countries of the authors, and reference lists, was searched, and any literature identified until September 2021 was included in this study. The purpose of this study was to evaluate the current status of research on TMM worldwide; therefore, reports, books, or journals that were available only in Mongolia were not included. The search strategy for the WoS is as follows.

  #1 traditional Mongolian medicine
  #2 TMM and Mongol
  #3 Mongolian medicine
  #4 #1 OR #2 OR #3

The following inclusion criteria were adopted for this bibliometric analysis:

- Literature type: all research articles (papers), conference proceedings, or data papers, regardless of the sources of the literature, were included in this study. No limitations were imposed in terms of the type of literature if it was searched in the WoS database. There were no linguistic limitations in this study.
- Publication period: all literature between the years 1991 and 2021 was included.
- Topics of the literature: any literature that dealt with research on TMM was included in this study. TMM was defined as “medicinal practice which is indigenous to Mongolian population and was established based on the tradition and beliefs of Mongolian people,” according to the explanation of MeSH terminology in PubMed [8]. Any research types including cell or animal experiments of various interventions of TMM, clinical studies, epidemiologic studies, reviews, and surveys of TMM practitioners were included in this study. Studies that were not related to TMM were excluded from analysis.

The titles and abstracts of each article were reviewed by two authors. Full texts were also assessed if there was insufficient information regarding the selection process. Two authors (T-HK and JWK) screened the articles individually, and decisions were made through discussion.

### 2.2. Data Extraction and Bibliometric Analysis Method

Bibliometric data from the included publications were retrieved from the WoS database. Full records with cited reference lists for each publication were exported as a plain text file that provided detailed information on all the included documents such as authors, document titles, publication source (or journal title), document types, keywords of the publications, KeyWords Plus®, which is a specific index term for each document and is generated using the title of the article by the WoS, country of the authors, abstract, year of publication, and reference lists [9]. The type of studies, number of authors for each document, country of the corresponding authors, most relevant affiliations, most cited documents, and sources (journal) of the documents were analyzed.

To assess trends in TMM research, authors' keywords and KeyWords Plus® were analyzed in a descriptive way. A thematic evolution map using keywords was suggested to present changes in research topics according to temporal changes. Coword analysis on the keywords of different documents was presented in a low-dimensional space based on the different periods of each publication, which was based on Cobo's method [10].

To analyze the research network among co-authors, affiliations, or countries of the authors, collaboration networks were obtained using the following formula:

Bcoll = *A*' *x A*, where *A* is a document *x* author matrix in the author collaboration network, a document *x* affiliation matrix in the affiliation collaboration network, and a document × country matrix in the country collaboration network. Each element in the matrix suggests the number of collaborations between two authors (institutions or countries) among the included publications [9,11]. The closeness centrality of each node was calculated by measuring the total distance from other nodes in the collaboration network plot, and the betweenness centrality was calculated by assessing the number of shortest paths from other nodes to a specific node [12]. The Bibliometrix package for R (Ver 3.1) was used in this bibliometric analysis.

## 3. Results

### 3.1. Summary of the Included Documents

**Figure 1:**
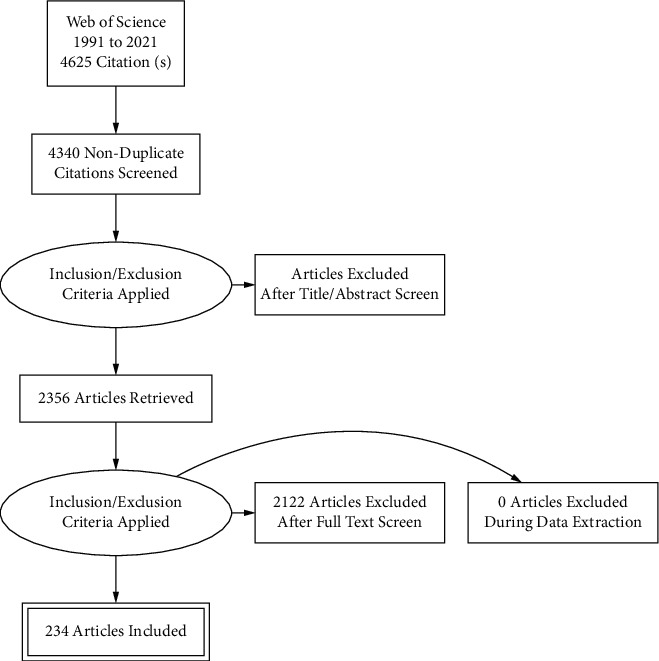
Study flow diagram.

From the 4,625 records identified from the WoS search, only 234 documents were included in this study (Figure 1). The average time from publication to 2021 was 5.68 years, and the average number of citations per document was 6.363. The total number of references was 6,426, and the number of all authors was 1,050. The number of co-authors per documents was 6.28. The most common types of documents such as articles (*n* = 202), reviews (*n* = 11), meeting abstracts (*n* = 13), editorial materials (*n* = 2), and notes (*n* = 1) were followed (Supplementary file 1). The number of annual publications has been increased gradually between 1991 and 2021 (Supplementary file 2). The number of average total article citations per year also showed gradual increase between 1991 and 2021 (Supplementary file 3).

### 3.2. Analysis of the Authors

Among the included 6,407 authors, Wang Q. H. (number of documents = 19), Batkhuu J. (number of documents = 10), Bao L. D. (number of documents = 9), and Glasl S. (number of documents = 9) were the most active authors and among the top 4 (Supplementary file 4). The most cited authors were Glasl S. (number of documents = 20), Kletter C. (number of documents = 19), Narantuya S. (number of documents = 19), Purevsuren S. (number of documents = 17), and Obmann A. (number of documents = 16). The most influential authors with the highest *h*-index were Glasl S. (7), Narantuya S. (7), Purevsuren S. (7), Batkhuu J. (6), Kletter C. (6), and Zehl M. (6) (Table 1).

### 3.3. Analysis of the Institutions and Countries

Institutions that included the most active authors in this study were identified, and Inner Mongolia Medical University was the most relevant one (*n* = 71). The Inner Mongolia University of Nationalities came second (*n* = 61), and the National University of Mongolia was the third institution (*n* = 16) (Table 2). Countries where the corresponding authors were from were analyzed, and the top five countries of the corresponding authors were China (*n* = 153), Japan (*n* = 28), South Korea (*n* = 9), Austria (*n* = 8), and Russia (*n* = 7). Mongolia had only 4 corresponding authors (Table 3).

### 3.4. Analysis of Journals and Cited Documents

The five most relevant journals that published documents related to TMM were the Journal of Ethnopharmacology (*n* = 24), Spectroscopy and Spectral Analysis (*n* = 16), Natural Product Research (*n* = 15), Journal of Natural Medicine (*n* = 8), Evidence-Based Complementary and Alternative Medicine (*n* = 6), and Planta Medica (*n* = 6) (Supplementary file 5). The most frequently cited document was “Protective effects of probiotic *Lactobacillus casei* Zhang against endotoxin and d-galactosamine-induced liver injury in rats via antioxidative and anti-inflammatory capacities” by Wang 2013 (Supplementary file 6) [13].

### 3.5. Analysis of Research Topic

**Figure 2:**
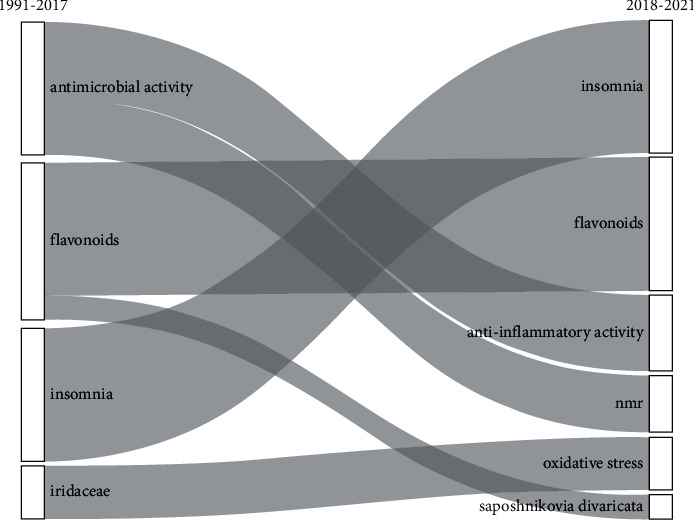
The thematic evolution map.

When the frequency of the author's keywords in each document was assessed, the most frequently used keywords were “flavonoids” (*n* = 12), “cytotoxicity” (*n* = 8), “NMR” (*n* = 8), “Tibetan medicine” (*n* = 7), “insomnia” (*n* = 6), and “trace elements” (*n* = 6). The most frequently used KeyWords Plus® were “constituents” (*n* = 17), “expression” (*n* = 16), “activation” (*n* = 15), “extract” (*n* = 11), and “inhibition” (*n* = 11) (Table 4). From the thematic evolution map, through keywords analysis, research topics changed from “antimicrobial activity,” “flavonoids,” “insomnia,” and “Iridaceae” (1991–2017) to “insomnia,” “flavonoids,” “anti-inflammatory activity,” “NMR,” “oxidative stress,” and “*Saposhnikovia divaricata*” (Figure 2). When assessing the network of keywords used in the publications, prominent co-occurrence was observed among different keywords such as “flavonoids” and “chemical constituents,” “NMR” and “Chinese minority traditional medicine,” and “Tibetan medicine” and “Warm acupuncture” (Supplementary file 7).

### 3.6. Collaboration Networks for Co-Authors, Institutions, and Countries

In the collaboration network of the authors, Wang QH showed the highest betweenness centrality (score = 17), but most authors exhibited very low betweenness centrality. On the closeness centrality score, most authors showed similar closeness centrality (Supplementary file 8). In the collaboration network of the institutions, Inner Mongolia University (score = 66), National University of Mongolia (score = 61), and Peking University (score = 56) showed the highest betweenness centrality among the included institutions (Supplementary file 9). However, closeness centrality had similar scores among the institutions. From these results, a very loose collaboration between authors can be identified. In the collaboration network of the countries, Mongolia and China showed the highest betweenness centrality (score = 15.82 in Mongolia and 1.18 in China) and closeness centrality (score = 0.14 in Mongolia and 0.11 in China). Therefore, the two countries can be assumed to be the core of the collaboration network in TMM research (Supplementary file 10).

## 4. Discussion

In this study, only a small number of research publications were identified through the WoS search (*n* = 229). The leading countries that the corresponding authors belonged to were China, Japan, and South Korea. From the analysis of research collaboration among countries, Mongolia and China showed the highest centrality scores for betweenness and closeness. The most actively used keywords were “flavonoids,” “cytotoxicity,” and “NMR.” Research topics from the included publications were mainly related to the chemical analysis of medicinal herbs or herbal products. Nonpharmacological interventions or TMM diagnostics have not been frequently evaluated.

This is the first bibliometric analysis on TMM in a global database, the WoS. An extensive search of literature on TMM was conducted, and appropriate publications were included based on predefined selection criteria. Although qualitative analysis of publication status was the main research methodology, collaboration networks were accessed by calculating the betweenness centrality and closeness centrality and suggested a thematically changing map through a coword analysis of keywords. The results of this study can provide a brief map of the current status of research on TMM among the publications available worldwide.

This study has several limitations that originate from the research methodologies used. First, the WoS was used only to search for relevant publications and extract data. The WoS is a widely used database that covers research publications and citations [14]. Because the WoS supplies various metadata, including document information (title and publication date), author information (author's name and information), and citation information (reference lists and citation numbers), users can export these metadata, which enables researchers to easily conduct a bibliometric analysis [15]. However, empirical evidence suggests that the WoS database has considerable errors that originate from the transcription procedure, such as mistyping or omission of authors and titles or incomplete (omitted) cited reference lists. In addition, online publications can introduce errors, which can double count the same publication or reduce the time it takes for a study to be published after being accepted, possibly distorting the influence on the citation of the study [16]. Another issue is related to the limited range of journals included in the WoS database [17]. From this perspective, it is acknowledged that there may have been errors in the selection and analysis of literature. Second, the search strategy may have been biased. Keywords such as “Traditional Mongolian medicine” and “Mongolian medicine” were used to identify publications in the WoS. If any document that had been classified as TMM research did not use these keywords in the title, abstract, or keywords, the search could not locate these publications. Although TMM is included in the MeSH's vocabulary, the omission of keywords or phrases allows for a limited literature search in PubMed, which is another reason why a specially developed search filter or strategy for TMM is necessary in the future [7].

From this study, it was found that the results of pharmacological studies have only been published actively by researchers in China and Mongolia. Like acupuncture in traditional Chinese medicine and Korean medicine, TMM has a variety of nonpharmacological interventions such as bloodletting therapy [18] and moxibustion therapy [19]. It has principles in the physiology and pathology of medical theory, distinguished from traditional medicine in other neighboring countries, which offers a different picture or clinical practice in TMM [20]. Therefore, balanced research on the various components of TMM should be encouraged.

## 5. Conclusion

There are not many publications on TMM, and research topics focus on the mechanism studies of herbs or botanical products in TMM. Nonpharmacological interventions or TMM diagnostics should be promoted in future studies. In addition, global research collaboration needs to be encouraged beyond China and Mongolia.

## Figures and Tables

**Table 1:** Top 10 most cited authors.

Author	Local citations^*∗*^	*H*-index
Glasl S.	20	7
Kletter C.	19	6
Narantuya S.	19	7
Purevsuren S.	17	7
Obmann A.	16	5
Batkhuu J.	14	6
Zehl M.	13	6
Murata T.	12	5
Sasaki K.	11	4
Suganuma K.	11	4

^*∗*^Local citations were counted from the included references.

**Table 2:** Most relevant affiliations (top 20 institutions).

Affiliations	Articles
Inner Mongolia Medical University	71
Inner Mongolia University of Nationalities	63
National University of Mongolia	18
Mongolian National University of Medical Science	15
Obihiro University of Agriculture and Veterinary Medicine	15
Baotou Medical College	14
Minzu University of China	14
Beijing University of Chinese Medicine	13
Institute of Chemistry and Chemical Technology	12
Inner Mongolia Normal University	11
University of Vienna	10
Health Sciences University of Mongolia	9
Taipei Medical University	9
Inner Mongolia University	8
Inner Mongolia Agricultural University	7
Institute for Mongolian Buddhist and Tibetan Studies	7
Jilin University	7
Peking University	7
Tohoku Medical and Pharmaceutical University	7
Shenyang Pharmaceutical University	6

**Table 3:** List of the corresponding authors' countries.

Country	Articles (%)
China	153 (68)
Japan	28 (12.4)
South Korea	9 (4)
Austria	8 (3.6)
Russia	7 (3.1)
Germany	5 (2.2)
Mongolia	4 (1.8)
USA	4 (1.8)
Australia	1 (0.4)
Canada	1 (0.4)
Hungary	1 (0.4)
India	1 (0.4)
Spain	1 (0.4)
Sweden	1 (0.4)
Thailand	1 (0.4)

**Table 4:** Most frequently used keywords and KeyWords Plus® (top 20).

Words assessed by authors' keywords	Occurrences	Words assessed by KeyWords Plus	Occurrences
Flavonoids	12	Constituents	17
Cytotoxicity	8	Expression	16
NMR	8	Activation	15
Tibetan medicine	7	Extract	11
Insomnia	6	Inhibition	11
Trace elements	6	Acid	10
ICP-AES	5	Apoptosis	10
Oxidative stress	5	Derivatives	9
*Syringa pinnatifolia*	5	Identification	9
Traditional medicine	5	Glycosides	8
Apoptosis	4	Oxidative stress	8
*Dianthus versicolor*	4	Rats	8
Hyperlipidemia	4	Mechanisms	7
Sesquiterpene	4	Cells	6
Spectroscopic methods	4	Extracts	6
Warm acupuncture	4	Flavonoids	6
Anti-inflammatory	3	Mice	6
Anti-inflammatory activity	3	Pathway	6
Antimicrobial activity	3	Roots	6
Antioxidant activity	3	Cancer	5

## Data Availability

The data used to support the findings of this study are available from the corresponding author upon request.

## Supplementary Materials

Supplementary Materials Supplementary file 1. Summary of the included research literature between 1991 and 2021. Supplementary file 2. Changes in the number of publications by year. Supplementary file 3. Average total article citations per year. Supplementary file 4. Most active authors (top 20). Supplementary file 5. Most relevant journal lists (top 20). Supplementary file 6. Most global cited documents. Supplementary file 7. Co-occurrence network of keywords. Supplementary file 8. Collaboration network of the authors in the literatures. Supplementary file 9. Collaboration network of the institutions in the literatures. Supplementary file 10. Collaboration network between countries.
