# Supplementary material for: Research Status Quo in Traditional Mongolian Medicine: A Bibliometric Analysis on Research Documents in the Web of Science Database
Source: Evid Based Complement Alternat Med. 2021 Dec 28;2021:5088129. doi: 10.1155/2021/5088129 (PMC8727094; doi:10.1155/2021/5088129)

Supplementary file 1. Summary of the included research literature between 1991 and 2021

**Items**	**Main information data (Total number of documents: 234)**
Average time duration from publication (mean)	5.68 years
Average numbers of citation per document	6.363
Total number of the references	6426
Document types	
Article	202
Editorial material	2
Meeting abstract	13
Note	1
Review	11
Number of all the authors	1050
Number of author's keywords	824
Number of the co-authors per documents	6.28
Collaboration index	4.64

Supplementary file 2. changes in the number of publications by year

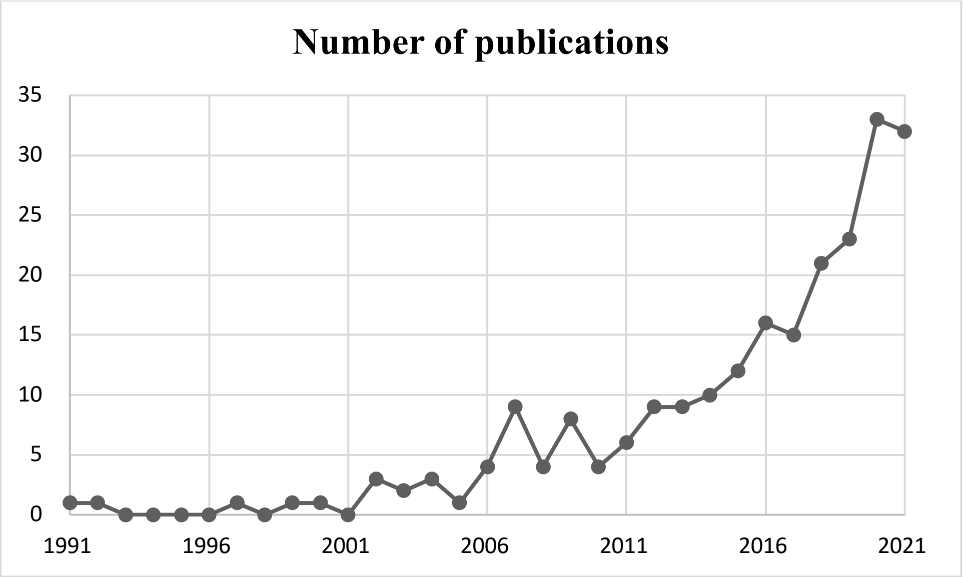

Supplementary file 3. Average total article citations per year

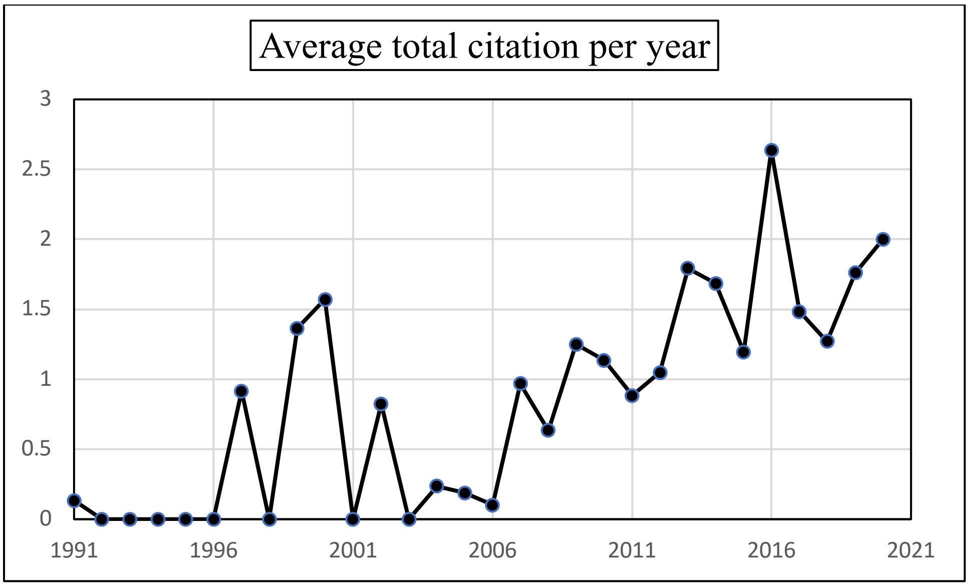

Supplementary file 4. Most active authors (top 20)

Authors	Articles
WANG QH	19
BATKHUU J	11
BAO LD	9
GLASL S	9
PUREVSUREN S	8
BO A	7
KLETTER C	7
LI MH	7
MURATA T	7
NARANTUYA S	7
SI LG	7
TU PF	7
AO WLJ	6
CHAI XY	6
OBMANN A	6
XUE PF	6
ZEHL M	6
BU R	5
KOMATSU K	5
LU JK	5

Supplementary file 5. Most relevant journal lists (top 20)

Sources	Articles
JOURNAL OF ETHNOPHARMACOLOGY	24
SPECTROSCOPY AND SPECTRAL ANALYSIS	16
NATURAL PRODUCT RESEARCH	15
JOURNAL OF NATURAL MEDICINES	8
EVIDENCE-BASED COMPLEMENTARY AND ALTERNATIVE MEDICINE	6
PLANTA MEDICA	6
CHEMICAL & PHARMACEUTICAL BULLETIN	4
MOLECULES	4
SCIENTIFIC REPORTS	4
ACTA PHARMACOLOGICA SINICA	3
CHEMISTRY OF NATURAL COMPOUNDS	3
CHINESE JOURNAL OF NATURAL MEDICINES	3
HERALD OF AN ARCHIVIST	3
JOURNAL OF NATURAL PRODUCTS	3
JOURNAL OF SEPARATION SCIENCE	3
JOURNAL OF TRADITIONAL CHINESE MEDICINE	3
PHARMACEUTICAL BIOLOGY	3
PHYTOCHEMISTRY	3
PHYTOCHEMISTRY LETTERS	3
BASIC & CLINICAL PHARMACOLOGY & TOXICOLOGY	2

Supplementary file 6. Most global cited documents

Document information	DOI	Total Citations
WANG YZ, 2013, INT IMMUNOPHARMACOL	Protective effects of probiotic Lactobacillus casei Zhang against endotoxin- and d-galactosamine-induced liver injury in rats via anti-oxidative and anti-inflammatory capacities	61
BONDIA-PONS I, 2014, FOOD RES INT	Metabolic profiling of Goji berry extracts for discrimination of geographical origin by non-targeted liquid chromatography coupled to quadrupole time-of-flight mass spectrometry	58
MENG QX, 2009, J ETHNOPHARMACOL	Ethnobotany, phytochemistry and pharmacology of the genus *Caragana*used in traditional Chinese medicine	58
MENG XX, 2016, J ETHNOPHARMACOL	Chemical composition, antibacterial activity and related mechanism of the essential oil from the leaves of *Juniperus rigida* Sieb. et Zucc against *Klebsiella pneumoniae*	47
TSEVEGSUREN N, 2007, J NAT PROD	Biologically Active Natural Products from Mongolian Medicinal Plants Scorzonera divaricata and Scorzonera pseudodivaricata	46
BAO B, 2007, MAR DRUGS	Monoindole Alkaloids from a Marine Sponge Spongosorites sp.	36
CHIK WI, 2015, J ETHNOPHARMACOL	*Saussurea involucrata*: A review of the botany, phytochemistry and ethnopharmacology of a rare traditional herbal medicine	35
KOJIMA K, 2000, CHEM PHARM BULL	Sesquiterpenoid derivatives from Ferula ferulioides. IV	33
TANAKA K, 2008, PHYTOCHEMISTRY	Quality evaluation of Astragali Radix using a multivariate statistical approach	33
KOJIMA K, 1999, CHEM PHARM BULL	Sesquiterpenoid derivatives from Ferula ferulioides. III	30
LI YJ, 2016, NEUROSCI LETT	*Xanthoceras sorbifolia* extracts ameliorate dendritic spine deficiency and cognitive decline via upregulation of BDNF expression in a rat model of Alzheimer's disease	28
MA CM, 2002, PHYTOTHER RES	Screening of Chinese and Mongolian herbal drugs for anti-human immunodeficiency virus type 1 (HIV-1) activity	26
ZHANG W, 2017, FOOD FUNCT	Seabuckthorn berry polysaccharide protects against carbon tetrachloride-induced hepatotoxicity in mice *via*anti-oxidative and anti-inflammatory activities	26
ROEDER E, 2009, PHARMAZIE	Pyrrolizidine alkaloids in medicinal plants of Mongolia, Nepal and Tibet	25
SCHMIDT S, 2013, J ETHNOPHARMACOL	Extracts from *Leonurus sibiricus* L. increase insulin secretion and proliferation of rat INS-1E insulinoma cells	23
CAO Y, 2016, J ETHNOPHARMACOL	Anti-myocardial ischemia effect of *Syringa pinnatifolia* Hemsl. by inhibiting expression of cyclooxygenase-1 and -2 in myocardial tissues of mice	22

KOJIMA K, 1997, PHYTOCHEMISTRY	Flavanones from *Iris tenuifolia*	22
LI MX, 2009, PHYTOTHER RES	Isolation and identification of hemostatic ingredients from lamiophlomis rotata (Benth.) kudo	22
BADRAL D, 2017, J NAT PROD	Flavonoid and Galloyl Glycosides Isolated from Saxifraga spinulosaand Their Antioxidative and Inhibitory Activities against Species That Cause Piroplasmosis	18
SONG SA, 2010, CHEM PHARM BULL	3-Hydroxymethylglutaryl Flavonol Glycosides from a Mongolian and Tibetan Medicine, Oxytropis racemosa	18

Supplementary file 7. co-occurrence network of keywords.

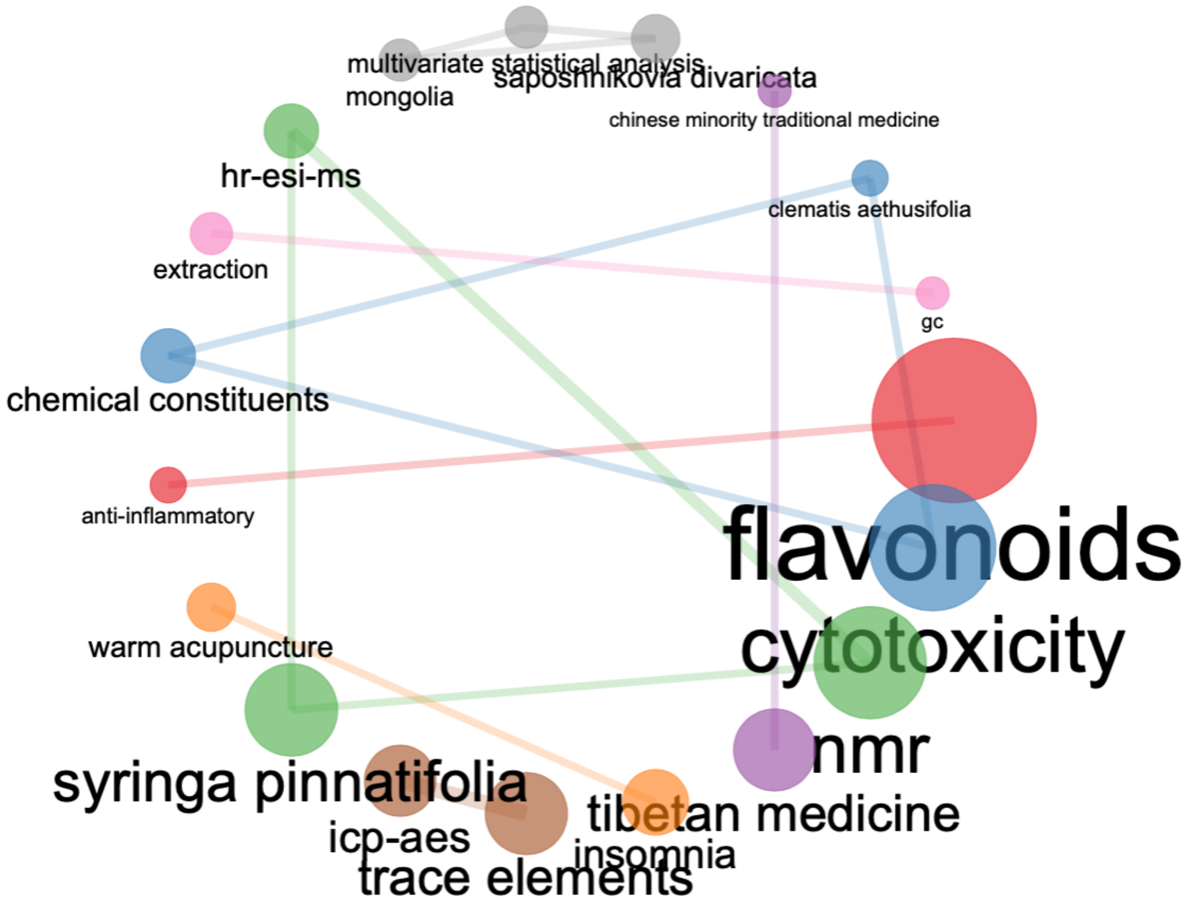

Supplementary file 8. Collaboration network of the authors in the literatures

Node	Cluster	Betweenness centrality	Closeness centrality
GLASL S	1	0	0.000659196
PUREVSUREN S	1	0	0.000659196
KLETTER C	1	0	0.000659196
NARANTUYA S	1	0	0.000659196
OBMANN A	1	0	0.000659196
ZEHL M	1	0	0.000659196
BAO LD	2	1.728571429	0.000677048
BO A	2	0	0.00067659
SI LG	2	2.271428571	0.000677048
YUAN HW	2	5	0.000677048
SU XL	2	0	0.000674764
WANG YH	2	0	0.00067659
CHEN YS	2	0	0.000675219
BATKHUU J	3	4	0.000659196
MURATA T	3	0	0.000658762
KOMATSU K	3	0	0.000657462
SASAKI K	3	0	0.000658762
SUGANUMA K	3	0	0.000658762
BANZRAGCHGARAV O	3	0	0.000658762
TU PF	4	0.321428571	0.000659196
CHAI XY	4	0.321428571	0.000659196
ZHANG RF	4	0.214285714	0.000659196
CAO Y	4	0.142857143	0.000659196
FENG X	4	0	0.000658762
GAO XL	4	0	0.000658762
XUE PF	5	0	0.000641849
BU R	5	0	0.000641849
LU JK	5	0	0.000641849
DONG X	5	0	0.000641849
MA FX	5	0	0.000641849
WANG QH	6	17	0.000696864
AO WLJ	6	0	0.000694444
BAO WQ	6	0	0.000694927
HAN JJ	6	0	0.000693963
PA B	6	0	0.000694927
XU YH	6	0	0.000694927
BAO XH	6	0	0.000694444
HAO JS	6	0	0.000693963
BORJIGIDAI A	7	0	0.000594884
CUI J	7	0	0.000594884
BA T	8	0	0.000594884

Supplementary file 9. Collaboration network of the institutions in the literatures

Node	Cluster	Betweenness	Closeness
UNIV VIENNA	1	0	0.00094697
HLTH SCI UNIV MONGOLIA	1	0	0.00094697
MED UNIV VIENNA	1	0	0.00094697
INNER MONGOLIA MED UNIV	2	66	0.001550388
INNER MONGOLIA UNIV NATIONALITES	2	38	0.001533742
MINZU UNIV CHINA	2	14	0.001506024
BEIJING UNIV CHINESE MED	2	14	0.00152207
JILIN UNIV	2	0	0.001501502
PEKING UNIV	2	56	0.001550388
INNER MONGOLIA INT MONGOLIAN HOSP	2	0	0.001526718
ALASHAN MONGOLIAN HOSPITAL	2	0	0.001490313
INST CHINESE MAT MED	2	0	0.001474926
NATL UNIV MONGOLIA	3	61.19920635	0.001545595
OBIHIRO UNIV AGR AND VET MED	3	1.467460317	0.00152207
INST CHEM AND TECHNOL	3	0	0.001517451
TOHOKU MED AND PHARMACEUT UNIV	3	0.333333333	0.001519757
MONGOLIAN UNIV LIFE SCI	3	0	0.001515152
TOYAMA UNIV	3	0	0.001512859
MONGOLIAN UNIV PHARMACEUT SCI	3	0	0.001517451
BAOTOU MED COLL	4	0	0.000918274
INNER MONGOLIA INST TRADIT CHINESE MED	4	0	0.000918274
INNER MONGOLIA AGR UNIV	5	0	0.000918274
INNER MONGOLIA PEOPLES HOSP	5	0	0.000918274
INNER MONGOLIA NORMAL UNIV	6	0	0.000918274
CHINESE AND MONGOLIAN HOSP INNER MONGOLIA	6	0	0.000918274
INNER MONGOLIA UNIV	7	0	0.000918274
INT HOSP MONGOLIAN MED	7	0	0.000918274
MONGOLIAN PHARMACEUT PREPARAT CTR	8	0	0.00094697
MONGOLIAN MED INST INNER MONGOLIA	8	0	0.00094697
STATE CLIN TRIAL INST NEW DRUGS	8	0	0.00094697
INST CHEM	9	0	0.000918274

Supplementary file 10. Collaboration network between countries

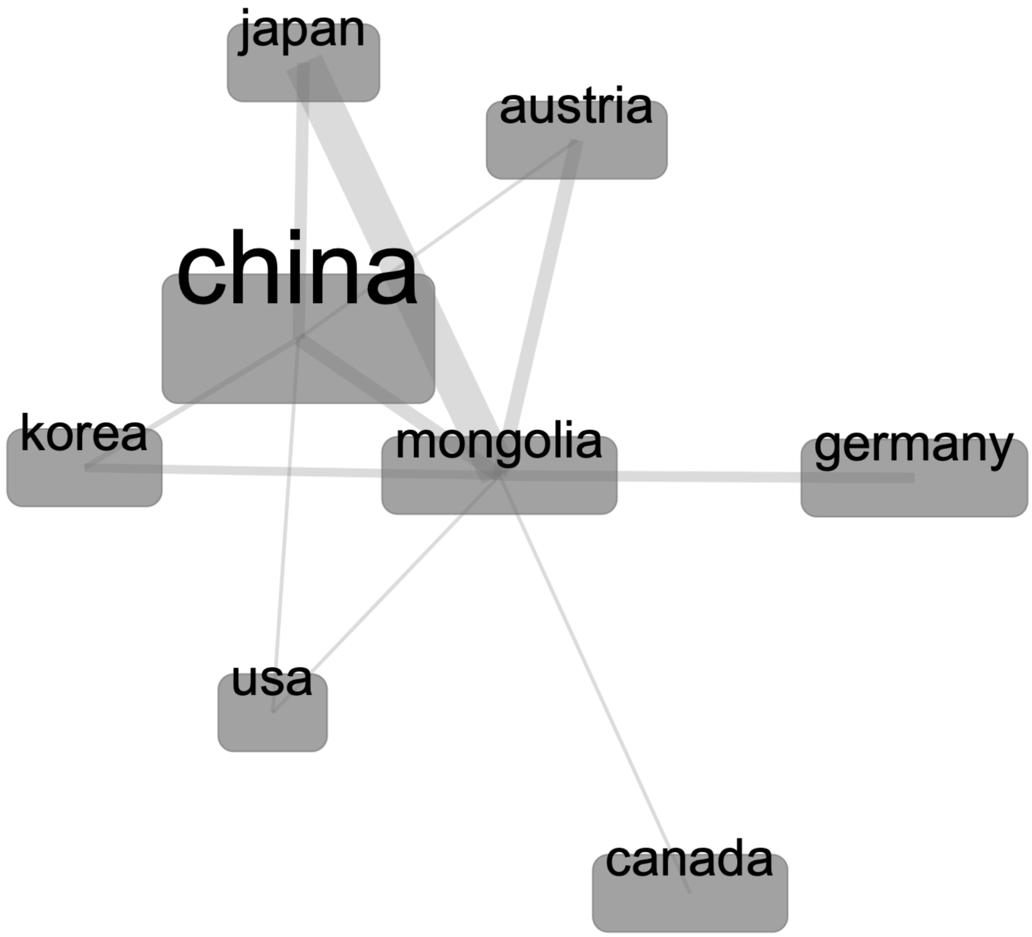

Supplement: Supplementary Materials — Supplementary file 1. Summary of the included research literature between 1991 and 2021. Supplementary file 2. Changes in the number of publications by year. Supplementary file 3. Average total article citations per year. Supplementary file 4. Most active authors (top 20). Supplementary file 5. Most relevant journal lists (top 20). Supplementary file 6. Most global cited documents. Supplementary file 7. Co-occurrence network of keywords. Supplementary file 8. Collaboration network of the authors in the literatures. Supplementary file 9. Collaboration network of the institutions in the literatures. Supplementary file 10. Collaboration network between countries. [file 5088129.f1.pdf]
